# Quantification of resting myocardial blood flow velocity in normal humans using real-time contrast echocardiography. A feasibility study

**DOI:** 10.1186/1476-7120-3-16

**Published:** 2005-06-16

**Authors:** Siri Malm, Sigmund Frigstad, Frode Helland, Kjetil Oye, Stig Slordahl, Terje Skjarpe

**Affiliations:** 1 Department of Circulation and Medical Imaging, Faculty of Medicine, Norwegian University of Science and Technology, Trondheim, Norway; 2 GE Healthcare Technologies, Ultrasound R&D, Trondheim, Norway

**Keywords:** Contrast echocardiography, myocardial perfusion, real-time imaging, contrast replenishment rate

## Abstract

**Background:**

Real-time myocardial contrast echocardiography (MCE) is a novel method for assessing myocardial perfusion. The aim of this study was to evaluate the feasibility of a very low-power real-time MCE for quantification of regional resting myocardial blood flow (MBF) velocity in normal human myocardium.

**Methods:**

Twenty study subjects with normal left ventricular (LV) wall motion and normal coronary arteries, underwent low-power real-time MCE based on color-coded pulse inversion Doppler. Standard apical LV views were acquired during constant IV. infusion of SonoVue^®^. Following transient microbubble destruction, the contrast replenishment rate (β), reflecting MBF velocity, was derived by plotting signal intensity vs. time and fitting data to the exponential function; y (t) =A (1-e^-β(t-t0)^) + C.

**Results:**

Quantification was feasible in 82%, 49% and 63% of four-chamber, two-chamber and apical long-axis view segments, respectively. The LAD (left anterior descending artery) and RCA (right coronary artery) territories could potentially be evaluated in most, but contrast detection in the LCx (left circumflex artery) bed was poor. Depending on localisation and which frames to be analysed, mean values of  were 0.21–0.69 s^-1^, with higher values in medial than lateral, and in basal compared to apical regions of scan plane (p = 0.03 and p < 0.01). Higher β-values were obtained from end-diastole than end-systole (p < 0.001), values from all-frames analysis lying between.

**Conclusion:**

Low-power real-time MCE did have the potential to give contrast enhancement for quantification of resting regional MBF velocity. However, the technique is difficult and subjected to several limitations. Significant variability in β suggests that this parameter is best suited for with-in patient changes, comparing values of stress studies to baseline.

## Background

MCE is an emerging technique for non-invasive evaluation of myocardial perfusion and coronary heart disease (CAD) [1-11]. Recent advances in multipulse technology have made real-time MCE feasible with low acoustic power [12-17], giving minimal contrast destruction and frame rates that facilitate evaluation of scan plane and wall motion. However, technical difficulties concerning tailored ultrasound equipment, imaging techniques, data-analysis and interpretation still remain to be solved.

The majority of MCE studies have reported data relying on visual assessment somewhat limited by its subjective approach [18]. Wei and coworkers pioneered a method for more objective quantification of MBF with contrast microbubbles administered as constant intravenous infusion [5]. From the time course of video intensity during progressively prolonged pulsing intervals, both MBF velocity and myocardial blood volume (MBV) could be assessed. The product of these two parameters was shown to correlate well with radiolabeled microsphere-derived MBF [5,17,19,20]. This quantitative approach has also been applied to real-time MCE techniques [14-16,19]. A strong linear correlation between the rate of signal intensity (SI) rise and volumetric flow has been reported, both at rest and during hyperemia [14,16,22]. On the other hand, steady state SI has not been found to correlate as well with flow measurements [14,17], indicating that the microbubble replenishment rate might be the major MCE perfusion parameter.

The quantification of replenishment rates is often limited to selected myocardial regions due to imaging problems [19,22]. To our knowledge there are limited human studies reporting resting replenishment rates for all standard myocardial segments measured in different cardiac phases. The aim of this study was 1) to evaluate the feasibility of a very low-power real-time MCE technique for visualising the perfusion in normal human myocardium, and 2) to quantify the MBF velocity, β, of all myocardial segments of the apical scan views by using the destruction-replenishment approach.

## Methods

### Study subjects

Twenty study subjects were enrolled; ten healthy male volunteers (age 24 ± 3) and ten patients (age 55 ± 5), five of them female. The study subjects were not screened for echocardiographic image quality, the only inclusion criteria being an age above 18 and confirmed normal left ventricular regional and global systolic function by conventional echocardiography. The ten patients had undergone coronary angiography due to chest pain, with the findings of open and normal coronary arteries and normal left ventricular end-diastolic pressures. The healthy volunteers were assumed to have normal coronary anatomy and myocardial perfusion, due to the abscence of CAD risk factors and symptoms, and normal findings on standard echocardiography. All the subjects were in sinus rhythm. Exclusion criteria were pregnancy or lactation, known allergy to the contrast agent, significant valve diseases or shunts, severe pulmonary hypertension, and severe extra-cardiac disease. All the subjects gave their written informed consent to the participation. The study conformed to the declaration of Helsinki, and the Regional Commitee of Medical Ethics approved the protocol.

### Contrast agent

The ultrasound contrast agent SonoVue^® ^(Bracco, Milan, Italy) was used, consisting of microspheres of sulphur hexafluoride gas (SF_6_) stabilised by a phospholipid monolayer in an aqueous solution. SonoVue^® ^was infused continuously by a manually rotated volume pump through a 20G vial in a proximal forearm vein. There were slight individual changes of the infusion rate (70–100 ml/ hour) to optimize the myocardial opacification and minimize the far-field attenuation. Once steady state was reached and the recording started, the infusion rate was held constant in every individual study.

### MCE technique

Imaging was performed with Vivid 7™ (GE Vingmed Ultrasound, Horten, Norway) with a M3S matrix array transducer. The contrast-specific application, Coded Harmonic Angio™, is a very low power, real-time technique based on pulse inversion combined with power Doppler, operating at a frame rate of 20 Hz. With this choice of application and contrast agent, the optimal agent-tissue-ratio was achieved with a mechanical index (MI) as low as 0.04–0.05. The signal amplitudes were color-coded by the Angio mode and displayed as overlays on fundamental tissue grey-scale images. The focus was set basally, close to the mitral valve plane. The depth was set to let the left ventricle fill the image sector, and color gain was adjusted to reduce the signal-to-noise ratio to the point that hardly any noise was observed within tissue and cavity. The time gain compensation was adjusted to obtain homogenous SI and to reduce the noise from the myocardium, the epi-/ pericardium and the mitral valves. After the initial adjustments all settings were held constant in every individual study.

Baseline imaging was acquired in tissue harmonic mode for confirmation of normal anatomy and wall motion. MCE was performed in the apical four-chamber, two-chamber and long-axis views. Standard views were at times slightly modified, i.e. by centralising the lateral or anterior walls in the scan sector, to optimize the contrast detection and avoid attenuation and shadowing. When the myocardial contrast opacification reached a steady state, a 'flash' of 15 frames of high MI (1.0), timed to cover at least the entire systole, was applied for transient microbubble destruction. This was followed by immediate, automatic return to low MI continuous imaging of microbubble replenishment in end-expiration (See Additional file: 1 Movie demonstrating a real-time destruction-replenishment loop of the LV apical long-axis view). The procedure was repeated twice for every scan view. Fifteen cardiac cycles of every destruction-replenishment sequence, at least 10 after 'flash', were captured and stored digitally as raw-data.

### Image analysis

The MCE data were analysed off-line on a PC workstation. Analyses of the cineloops were performed blinded in random order using EchoPAC PC™ (GE Vingmed Ultrasound, Horten, Norway). Measurement of mean signal intensity (dB) was done in manually placed, equally sized and shaped regions of interest (ROI) in the 16 standard myocardial segments [23], plus the two apical segments of the apical long-axis view. The ROIs were large, avoiding high intensity signals from the cavity and the epi-and endocardium. When necessary, their position was slightly adjusted to compensate for the translation of the heart. The depth of the ROI position was not changed. Finally, all ROIs were 'anchored' for each frame.

The myocardial SI was plotted against time (t) and fitted to the exponential function: **y (t) =A (1-e^-β(t-t0)^) + C**, where **y **is SI at any time during the contrast replenishment, **A **is the plateau SI corresponding to MBV, **β **is the rate of SI rise reflecting the mean bubble velocity or MBF velocity, and **C **is the intercept at the origin reflecting the background intensity level [5]. The introduction of **t_0 _**simply reflects that the analysis software allowed one to choose where to set t = 0. To further compensate for a possible non-zero initial value after flash, the constant C was added, implicating that the curve fitting was relatively independent of background myocardial SI. The ROIs were positioned and anchored before the curve fitting was applied. Segmental values of A and β were derived from the replenishment cycles by careful frame-by-frame analysis. Separate quantitative analysis was performed both for all-frames, for selected end-systolic (end of T-waves) and end-diastolic (close to peak R-wave) frames.

**Figure 1:**
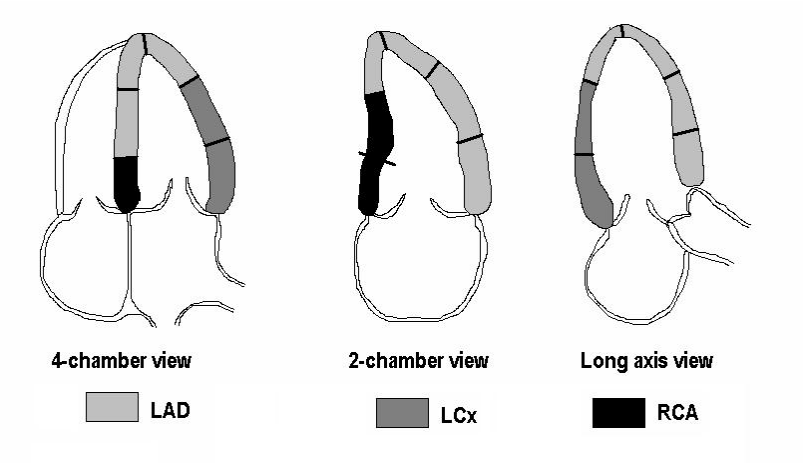
The different coronary artery beds and their representation in myocardial segments of the LV apical views, given a balanced coronary circulation. LAD = left anterior descending artery; LCx = left circumflex artery; LV = left ventricle; RCA = right coronary artery. *Courtesy of Asbjorn Stoylen, dept. of Circulation and Medical Imaging, Norwegian University of Science and Technology, Trondheim, Norway*.

The myocardial segments were assigned to the coronary artery perfusion territories (Figure 1), and the feasibility for evaluating perfusion at a territorial level was assessed. Because the LV wall motion was normal, any lack of myocardial contrast opacification was considered to be due to attenuation or inadequate detection, and the current segment was excluded from the quantitative analysis. Since the healthy volunteers all had normal regional and global LV function, it seemed acceptable to make this assumption even if coronary angiography was not performed.

### Statistics

Continuous variables are presented as mean ± 1SD. Comparison between groups was performed with linear regression analysis (ANOVA), a posthoc analysis was done using Bonferroni's correction. Differences were considered statistically significant at p less than 0.05 (two-sided) with a power of 0.80.

## Results

**Figure 2:**
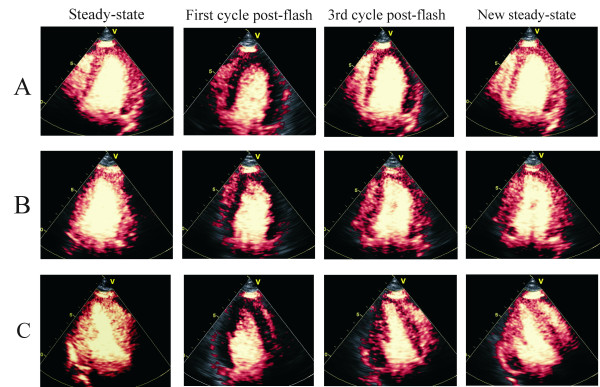
Some selected end-systolic images from destruction-replenishment sequences. A. 4-chamber view, B. 2-chamber view, C. Apical long-axis view.

Some visible myocardial contrast enhancement was obtained in all views of all the study subjects. Precontrast myocardial tissue SI was negligible, but in spite of careful adjustment of the time-gain compensation we noticed relatively strong precontrast signals from the mitral valve and basal epi-and pericardium. On average 6 minutes of infusion time was spent to aquire repeated replenishment cineloops in the apical views. Myocardial contrast first appeared around one minute after the infusion was started, and steady-state SI was reached after a mean period of 2.5 minutes. After the 'flash' an almost complete disappearance of myocardial color signals was observed, leaving the myocardium dark. Real-time visual grading of myocardial SI during post-destruction wash-in was difficult due to cardiac contraction, translation and cyclic changes of myocardial SI, both between systole and diastole and from beat to beat. By reviewing selected end-systolic frames, refilling was first observed in the mid-septum progressing to full opacification in 3 to 5 heartbeats. However, when observed in end-diastole the refilling clearly appeared faster, yet variable. Selected end-systolic images of destruction-refill sequences of the apical LV views are presented in Figure 2.

### Feasibility of quantitative analysis

All 360 myocardial segments were evaluated regardless of baseline image quality. Since the LV wall motion was normal, any contrast defect was considered to be due to attenuation or inadequate detection, and the current segment was thus excluded from the quantitative analysis. Following this, the myocardial opacification was regarded as sufficient for quantification in 98 of 120 (82%) of the four-chamber view segments (Table 1). The septum filled in completely during wash-in, while enhancement of the lateral wall was more pathcy. In two-chamber and long-axis views, the number of feasible segments was lower; 59 of 120 (49%) and 76 of 120 (63%), respectively (Table 1) Thus, a total of 233 of 360 (65%) of myocardial segments were feasible for quantification. For healthy, young normals and patients the feasibility was 118 (66%) and 115 (64%) of segments, respectively. The most frequent dropouts were observed in the mid and basal segments of the lateral and anterior wall, and in the basal segment of the inferolateral wall. In these we only obtained myocardial opacification in half of the study subjects.

**Table 1:** Absolute values of segmental myocardial contrast replenishment rate,  (s^-1^)

**Scan view**	**Myocardial segment**	**N**	**End-systolic analysis**	**End-diastolic analysis**	**All frames analysis**
**Apical Four-Chamber**	Basal septum	20	0.41 ± 0.11	0.63 ± 0.14	0.56 ± 0.17
	Mid septum	20	0.36 ± 0.12	0.46 ± 0.15	0.47 ± 0.16
	Apical septum	20	0.33 ± 0.14	0.43 ± 0.14	0.42 ± 0.18
	Apical lateral	18	0.29 ± 0.10	0.43 ± 0.18	0.30 ± 0.12
	Mid lateral	11	0.27 ± 0.11	0.44 ± 0.18	0.37 ± 0.12
	Basal lateral	9	0.32 ± 0.13	0.55 ± 0.12	0.37 ± 0.14

**Apical Two-chamber**	Basal inferior	14	0.43 ± 0.17	0.59 ± 0.19	0.44 ± 0.12
	Mid inferior	15	0.36 ± 0.09	0.54 ± 0.11	0.46 ± 0.15
	Apical inferior	13	0.35 ± 0.10	0.43 ± 0.14	0.34 ± 0.15
	Apical anterior	6	0.34 ± 0.10	0.45 ± 0.15	0.60 ± 0.29
	Mid anterior	5	0.27 ± 0.18	0.31 ± 0.18	0.21 ± 0.15
	Basal anterior	6	0.24 ± 0.21	0.39 ± 0.11	0.35 ± 0.22

**Apical Long-axis**	Basal inferolat.	7	0.42 ± 0.14	0.69 ± 0.12	0.61 ± 0.17
	Mid inferolat.	11	0.36 ± 0.15	0.52 ± 0.16	0.40 ± 0.18
	Apical inferolat.	13	0.31 ± 0.13	0.42 ± 0.18	0.44 ± 0.16
	Apical anterosept.	18	0.31 ± 0.11	0.48 ± 0.23	0.33 ± 0.15
	Mid anterosept.	15	0.36 ± 0.15	0.41 ± 0.19	0.47 ± 0.11
	Basal anterosept.	12	0.45 ± 0.19	0.58 ± 0.20	0.48 ± 0.16

Values are mean ± 1SD. N = number of study subjects in which myocardial contrast opacification of the current segment was regarded as feasible for quantification.

Evaluated on a territorial level, perfusion in the LAD area could be assessed in all 20 subjects. Segments usually assigned to the RCA could be evaluated in 15 subjects, whereas the LCx supply area was analysable in only half of the subjects.

### MCE parameters

**Figure 3:**
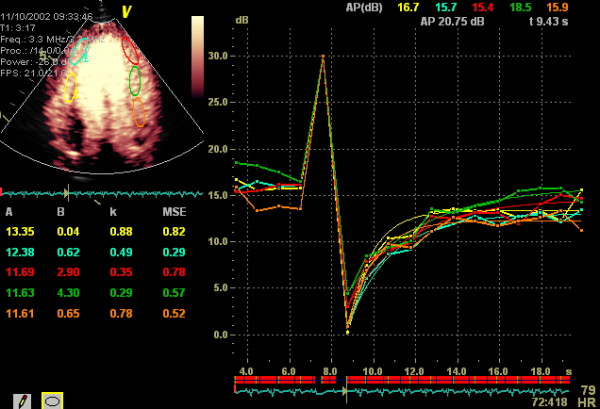
ECG-triggered end-systolic analysis (EchoPAC PC™) of contrast replenishment in the LV apical long-axis view. Time-intensity plots are fitted to the exponential function **A (1-e^-β(t-t0)^) + C. **Typical attenuation artefact is seen in basal inferolateral wall. A = peak plateau signal intensity reflecting myocardial blood volume; B(C) = intercept at the origin; k (β) = rate of signal intensity rise (microbubble replenishment rate) reflecting myocardial blood flow velocity; LV = left ventricle.

Beta-values were derived by curve fitting in the 233 feasible segments (Figure 3). Depending on localisation and which frames of the heart cycle to be analysed, we found mean values of β ranging from 0.21 to 0.69 s^-1 ^with SDs of 0.09 to 0.29 s^-1 ^(Table 1). Segmental mean values of A ranged from 6.01 to 12.29 dB with SDs of 2.1 to 4.9 dB. Mean end-systolic β-values was found to be higher in medial than lateral parts of the scan plane (0.37 ± 0.13 vs. 0.32 ± 0.14 s^-1^, p = 0.03), and at greater depths (basal; 0.45 ± 0.16 s^-1 ^vs. apical; 0.36 ± 0.14 s^-1^, p < 0.01). The A parameter similarly was found to be higher in medial (9.89 ± 2.7 dB) than lateral regions (7.99 ± 3.6 dB, p < 0.01), while it was significantly lower in basal than apical segments (7.87 ± 3.0 vs. 9.13 ± 2.74, p < 0.01).

By using the software's capability to perform an off-line ECG triggering, we did separate analysis from end-systolic and end-diastolic images (Figure 3). Significantly higher β-values were obtained when end-diastolic frames were analysed compared to end-systolic ones (0.49 ± 0.16 s^-1 ^and 0.35 ± 0.13 s^-1 ^respectively, p < 0.001). The β-values from all-frames analysis were lying between (0.43 ± 0.17 s^-1^), significantly different from both end-systolic and end-diastolic values (p = 0.002 and p = 0.001, respectively). Approximately the same level of differences was found between cardiac phases for A-values.

There were no significant differences in mean end-systolic β between four-chamber, two-chamber and long-axis views (0.34 ± 0.12 vs 0.35 ± 0.15 vs. 0.36 ± 0.14 s^-1^, respectively) nor between healthy volunteers and patients (0.36 ± 0.14 vs. 0.35 ± 0.13 s-1).

### Hemodynamic and safety parameters

There were no significant changes in the study subjects' blood pressure, heart rate or rythm during performance of the MCE examinations. Each subject received a total dose of 9.5 ml of SonoVue^®^, and none of them experienced any adverse effect in the observation period, nor were any observed.

## Discussion

Despite recent advances in contrast-specific imaging, our study demonstrates some of the difficulties with and the still limited ability of low-power real-time MCE for quantitative assessment of regional myocardial perfusion. The contrast detection was better in the segments with good precontrast myocardial image quality, the lateral and anterior walls being the poorest with frequent dropouts. The MCE imaging problems were reduced, but not eliminated by adjustment of infusion rates and by carefully repositioning the wall of interest more centrally in the scan sector. Nevertheless, two thirds of segments were feasible for quantitative analysis, and by assigning segments to the coronary territories, the LAD and RCA areas could potentially be evaluated. On the other hand, limited contrast detection made assessment of the LCx area difficult in more than half of the subjects.

Our study was not designed to test specific machine settings or examination variables. Nevertheless, we observed decreasing A values moving distally and laterally in the scan sector. β was similarly found to be lower in lateral than more axial parts, but in opposite to A it significantly increased at greater depths of the sector. This is in accordance with results from in vitro flow phantom and animal models [14,16], but has previously not been reported in human studies.

The exact mechanism for this spatial variability is uncertain. Variations in beam elevation and non-uniformity of the sound field are probably contributing factors [6,24,25]. With a phased array transducer, the acoustic energy delivered is lower laterally than in the centre of the sector, due to smaller effective aperture and some directivity of the elements [26]. Consequently, the backscatter from the microbubbles is inhomogenous. In addition attenuation and other artefacts decreasing backscatter are often more pronounced in the lateral regions. Non-uniformity of the effective regional 'flash' energy level might lead to variable bubble destruction, possibly altering the refilling parameters as well.

Far field attentuation likely contributed to the lowering of A in basal parts of sector. The finding of higher β in basal compared to apical segments could be due to the narrower beam elevation in the focal zone (which was set close to the mitral valve plane), resulting in a thinner scan plane with myocardial contrast destruction, and thereby faster replenishment of contrast from adjacent areas not affected by destruction.

The contrast refilling measurements were also influenced by the selection of frames from the cardiac cycle. We assessed significantly higher β-values from end-diastolic compared to end-systolic measurements. Corresponding values from all-frames analysis were lying in-between. Even the first diastolic frames after the 'flash' displayed some contrast signals, while the earliest systolic frame did not. This might be due to early bubble refilling through larger intramyocardial arterioles that are patent in diastole, but collapsed during systolic contraction [27,28]. By applying ROIs of uniform shape and size, the risk of capturing strong signals from the pericardium and the cavity seems to be greater in diastole, when the myocardium is thinner and localized more laterally in sector. Assessment of end-systolic frames seems preferable, despite more coronary flow in diastole, because the myocardium is thicker with less risk of 'contamination' from contrast in the blood pool.

These findings are in accordance with results from experimental studies [14,16]. Leong-Poi et al also found that MBF derived from images obtained in end-systole accurately reflected radiolabeled microsphere-derived MBF, whereas the correlation was poor, with significant overestimation of MBF, when the end-diastolic ones were used [16].

Insufficient contrast detection with too low agent-to-tissue signal ratio still remains a basic problem in quantifying the perfusion by pulse inversion Doppler. One disadvantage of the low-power real-time mode compared to destructive intermittent imaging, is the weaker backscatter from the non-destructive microbubble behaviour. A larger amount of contrast agent is required, which must be balanced against the degree of far field attenuation. Furthemore, some tissue harmonics are always present, and the movement artefacts will probably not be completely removed by the addition of power Doppler.

Relatively low temporal resolution was another anticipated problem. However, in our experience the applied frame rate of 20 Hz gave sufficient image quality to allow anatomical orientation and wall motion assessment.

An advantage of real-time MCE is the very short data aquisition time. Information that would require approximately a minute with intermittent imaging was recorded during seconds, making it possible to obtain all refill frames during one breathhold. The mean contrast infusion time of 6 minutes allowed us to aquire several replenishment loops from each scan view in every subject. But despite short data acquisition time, the procedure is still demanding. Given a beam elevation of only 3–4 mm at focus, it is mandatory to maintain a stable probe position during the entire destruction-replenishment period. Of the same reson, it is important to put an effort in avoiding off-axis views, particularly whenever tilting the scanplane to centralize a wall for improving contrast detection. Out-of plane imaging would possibly contribute to increased variability of the segmental quantitative parameters. Furtermore, the patients must be cooperative and able to hold their breath for up to ten seconds, preferably in end-expiration, to avoid lung interference and cardiac translation. Even some of our young, healthy study subjects experienced difficulties in this regard.

### Study limitations

The sample size of this study was small, and the patients were selected from baseline image quality. The coronary anatomy was not known in all study subjects, and we did not use other methods to ensure normal perfusion in our patients. However, due to the selection criteria it is highly unlikely that subclinical CAD could explain our results.

The main limitation of this study is that we did not apply any manipulations to change blood flow, i.e. adding of vasodilator to obtain maximal hyperaemia. Regional resting perfusion is normally variable, both between segments and study subjects, and this variability is further reinforced by imaging and technical problems with the applied destruction-replenishment approach. In the presence of a non-critical coronary stenosis, normal resting blood flow is maintained by arteriolar vasodilation. And patients with critical lesions often have collateral circulation, making assessment of resting perfusion complex. Hence, the interpretation of replenishment kinetics is particularly difficult at rest. Since normal perfusion is expected to increase at least 3-fold during hyperaemia, giving faster contrast replenishment after transient destruction, the evaluation of stress-to-rest ratios (the MBF velocity reserve) would be a more appropriate quantitative approach to assess normal or abnormal perfusion.

Interestingly, a simplified algorithm using qualitative evaluation of MBF velocity (actually time to complete myocardial opacification after destruction) from a single stress MCE perfusion study, was recently shown to detect CAD in patients with normal left ventricular function at rest, avoiding the need for resting MCE studies [11]. However, in that study triggered imaging was used, and it still seems to be a challenge maintaining a stable probe position for good image alignment during stress studies.

## Conclusion

Our study indicated that a very low-power real-time MCE could provide contrast opacification in multiple myocardial segments of the LV apical views. However, the acquistion of flash-replenishment loops adequate for quantification is limited by imaging and technical problems. The interpretation of regional resting replenishment curves is in addition complicated by great variability in normal perfusion. Our data support previous findings in experimental studies, which established that the absolute values of MBF velocity are highly influenced by ultrasound field geometry and which cardiac phase to be analyzed. The MBF velocity need to be interpreted with regard to the imaging technique used and the segments from which they are obtained, and due to the great variability is is probably best applied by analysis of relative changes, that is comparing values during stress with baseline.

## competing interests

The author(s) declare that they have no competing interests.

## Supplementary Material

Additional file 1 Movie demonstrating a real-time destruction-replenishment loop of the LV apical long-axis view Click here for file
